# Circadian coordination: understanding interplay between circadian clock and mitochondria

**DOI:** 10.1080/19768354.2024.2347503

**Published:** 2024-05-07

**Authors:** Jeongah Kim, Woong Sun

**Affiliations:** Department of Anatomy, College of Medicine, Korea University, Seoul, Korea

**Keywords:** Circadian rhythm, molecular clock, mitochondria

## Abstract

Biological rhythms play a crucial role in temporally regulating behavioral, physiological, and cellular processes within our bodies. One prominent example is the circadian rhythm, which enables our bodies to anticipate external cues and regulate our internal processes accordingly. The circadian rhythm is controlled by a molecular feedback loop known as the circadian clock, present in nearly all cells. The regulation of genes involved in mitochondrial function is no exception. Key aspects such as oxidative phosphorylation, mitochondrial biogenesis, and mitochondrial morphology are regulated by the circadian clock. Functional changes in mitochondria can retrogradely affect the circadian rhythm. Furthermore, there are also transcriptional circadian clock-independent rhythms within mitochondria. This review discusses mitochondrial rhythms independently or in communication with the circadian clock in the nucleus at the cellular level.

## 1. Introduction

Rhythm is an essential component of the biological system. Accordingly, many different forms of rhythmicity with a wide range of time scales have been discovered at the cellular, tissue, and organismal levels. Accumulating evidence supports the idea that various behavioral and physiological rhythms found in organisms are also controlled by molecular machineries governing rhythmicity via hormonal or neural controls. The best-known example of this is the molecular mechanisms of circadian control of animal behaviors. The same circadian rhythm-controlling machineries also regulate energy homeostasis, endocrine hormone levels, synaptic activity, and many other aspects of cellular functions (Herzog 2007; Gamble et al. 2014; Reinke and Asher 2019). Intriguingly, there is evidence that the circadian rhythm machinery also controls mitochondria-mediated biological functions (Manella and Asher 2016; Puig et al. 2018).

Genome-wide transcriptome investigations revealed that 43% of all protein-coding genes exhibited circadian oscillation in an organ-specific manner (Zhang et al. 2014). Additionally, proteome-wide studies revealed that protein abundances were also rhythmic, suggesting that the circadian clock regulates post-transcriptional processes (Reddy et al. 2006; Mauvoisin et al. 2014). Approximately 30% of circadian transcripts are affected by post-transcriptional regulation in mouse liver and drosophila head (Luck et al. 2014). Consequently, a wide variety of biological functions are under the regulation of the circadian clock, and mitochondrial functions are no exception (Manella and Asher 2016; Puig et al. 2018). Evidence suggests that mitochondrial metabolism is regulated by the circadian clock system through transcriptome, proteomics, and metabolomics studies (Masri et al. 2013; Kohsaka et al. 2014; Neufeld-Cohen et al. 2016; Mauvoisin et al. 2017). Furthermore, mitochondrial metabolism affects not only cellular metabolism but also the circadian clock, suggesting that communication between mitochondrial function and the circadian clock may play a pivotal role in regulating various physiologies and behaviors.

Considering that the circadian clock system is evolutionarily conserved, and mitochondria evolved from alpha-prokaryotes, there is also a possibility of the existence of circadian clock-independent rhythms within mitochondria. Therefore, there likely exist both independent or interdependent circadian and non-circadian rhythmic processes between these two cellular systems. In this review, we will discuss the circadian clock and mitochondrial rhythm and the potential interaction of these two rhythms at the cellular level.

## 2. Circadian rhythm

Circadian refers to a biological oscillation with a periodicity of roughly 24 h (circa diem, which is Latin for ‘about a day’). Organisms utilize internal timekeeping systems that synchronize with the day/night cycles, aligning with the solar movements around the planet during a 24-hour period to adapt and respond to the daily environmental cycles. The circadian clock, an internal timer, is present in almost all species, from cyanobacteria to humans. The molecular mechanism controlling the pacemaker is also well conserved (Takahashi 2017). In mammals, circadian clocks are thought to be hierarchically arranged, with a master clock in the hypothalamic suprachiasmatic nucleus (SCN) synchronizing peripheral clocks dispersed throughout the body with the help of endocrine and systemic inputs (Reppert and Weaver 2002).

**Figure 1.**
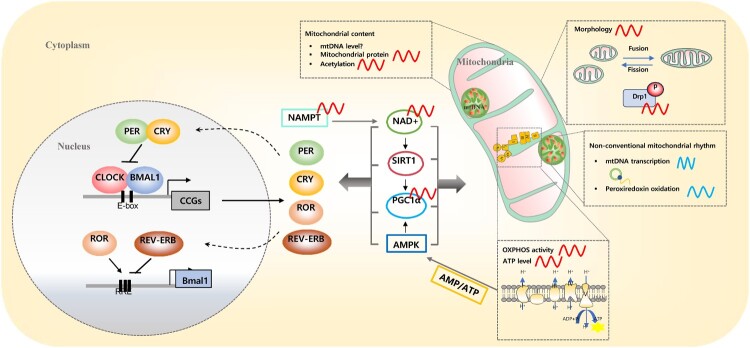
Schematic diagram of the circadian molecular clock and mitochondrial rhythm. The circadian clock consists of transcriptional translational feedback loops involving clock genes and proteins such as CLOCK, BMAL1, PER, and CRY. A secondary feedback loop is established by nuclear receptors such as ROR and REV-ERB, providing additional stability to the circadian oscillator. Circadian rhythm is important for regulating behavioral, physiological, and cellular processes in our bodies, and mitochondria are no exception. Various aspects of mitochondrial content, gene expression, morphology, and function exhibit circadian rhythms. The protein levels and acetylation of mitochondrial genes, as well as mitochondrial morphology and oxidative phosphorylation, are influenced by circadian rhythms. Additionally, there exist mitochondrial rhythms independent of the transcriptional circadian clock, such as the 12-hour ultradian rhythm of mtDNA transcripts and the circadian rhythm of peroxiredoxin oxidation, which regulates redox state even in red blood cells lacking a nucleus. While the circadian molecular clock impacts mitochondrial rhythms, changes in mitochondrial metabolism can retrogradely influence the molecular clock. The circadian clock-driven rhythmic expression of the rate-limiting enzyme NAMPT in NAD+ biosynthesis generates oscillations in NAD+ levels, thereby acting as a mediator regulating the circadian clock and mitochondrial contents and functions through the modulation of expressions such as SIRT1 and PGC1α. Additionally, AMPK functions as a nutrient sensor, connecting mitochondrial OXPHOS and the circadian clock. The reciprocal impact between mitochondrial rhythm and the circadian clock mechanisms is crucial for regulating diverse behaviors and physiological functions within our bodies.

Clock genes, which create transcriptional-translational feedback loops (TTFL), constitute the circadian clock in mammals (Figure 1). One loop depends on the proteins aryl hydrocarbon receptor nuclear translocator-like protein 1 (ARNTL1, also known as BMAL1), neuronal PAS domain protein 2 (NPAS2), and circadian locomotor output cycles kaput (CLOCK). BMAL1 combines CLOCK or NPAS2 to form CLOCK:BMAL1 or NPAS2:BMAL1 heterodimer, which activates the production of the repressor proteins Period (PER) and Cryptochrome (CRY) by binding to the E-box in the promoter regions of the nucleus. A new circadian cycle begins when the proteins PER and CRY are recruited into the nucleus and suppress CLOCK:BMAL. Nuclear receptors of RAR-related orphan receptor alpha (RORα) and nuclear receptor subfamily 1 group D (NR1D; also known as REV-ERBα) form a second feedback loop that adds more stability to the circadian oscillator. They are activated by the BMAL1/CLOCK heterodimer, and the binding of REV-ERBα and RORα to ROR response element (RORE) governs Bmal1 oscillation. Bmal1 expression is rhythmically activated by RORα, whereas Bmal1 transcription is inhibited by REV-ERBα. Notably, this traditional circadian clock machinery relies heavily on the transcriptional regulation of the downstream genes forming clock loops.

## 3. Mitochondria overview

From an evolutionary perspective, mitochondria are organelles that originated from alpha prokaryotes and have symbiotically evolved into cellular organelles. During evolution, most genes encoding mitochondrial proteins have transferred to the nucleus, but some have remained in the mitochondria, resulting in harboring their own genome. Human mitochondrial DNA (mtDNA) exists in a circular form consisting of 16,569 base pairs and encodes 13 mRNA, 2 rRNA, and 22 tRNA. mtDNA-encoded mRNA comprises OXPHOS complex proteins (Rackham and Filipovska 2022). Therefore, coordination between the nucleus and mitochondria is important for maintaining mitochondrial function.

Mitochondria served as central hubs regulating cellular metabolism, controlling important cellular processes such as ATP production through OXPHOS, and fatty acid synthesis. Additionally, they are involved in a variety of physiological processes including regulation of intracellular reactive oxygen species, calcium homeostasis, and apoptosis. To maintain mitochondrial quality and sustain mitochondrial function, mitochondrial morphological dynamics are crucial (Westermann 2010). Mitochondria are highly dynamic organelles, undergoing coordinated cycles of fusion and fission. Mitochondrial fission is essential for the elimination of dysfunctional or damaged mitochondria through the process of mitophagy. Mitophagy is crucial for mitochondrial quality control, and the maintenance of mitochondrial quality and quantity is ensured through mitophagy and mitochondrial biogenesis, which generate new mitochondria.

## 4. Circadian clock control of mitochondria

To adapt and respond to energy demands throughout the day, the metabolic hub, mitochondria, is influenced by the circadian clock (Figure 1). Many metabolic pathways exhibit pre-programmed activity according to the diurnal rhythm, which coincides with the feeding/fasting cycle (Asher and Schibler 2011). Proteomic analyses of isolated mitochondria by biochemical fractionation revealed that approximately 38% of mitochondrial annotated proteins exhibit a 24-hour periodicity of oscillation, suggesting that mitochondrial protein oscillation may affect mitochondrial function (Neufeld-Cohen et al. 2016). Chromatin immunoprecipitation (ChIP)-sequencing datasets of Bmal1, Clock, and Cry (Koike et al. 2012) revealed that the mitochondrion is a top cellular component, and 211 genes are related to mitochondrial function, including OXPHOS complex components, the TCA cycle, mitochondrial dynamics, β-oxidation, metabolite transporters, and apoptosis, suggesting that various functions of mitochondria are under circadian control (Jacobi et al. 2015). Also, transcriptome analyses show that Bmal1 deficiency alters cardiac energy metabolism involving fatty acid metabolism, glucose metabolism, the TCA cycle, and OXPHOS activity (Kohsaka et al. 2014). While the transcript levels of some nuclear-encoded mitochondrial proteins are dysregulated in Clock gene mutant mice (Kohsaka et al. 2014; Gong et al. 2015), a global analysis showed only a weak correlation between the phase of the mitochondrial proteome and its respective transcriptome, pointing towards the important involvement of post-transcriptional mechanisms in the circadian control of mitochondrial content (Neufeld-Cohen et al. 2016). It has also been suggested that post-translational changes have a significant impact on the dynamics and operations of mitochondria. Specifically, rhythmic acetylated proteins showed dependency of subcellular localization, with mitochondrial proteins being particularly enriched (Mauvoisin et al. 2017). A global analysis of the mouse liver’s acetylome found enrichment for CLOCK-dependent acetylation sites within the Krebs cycle and glutathione metabolism enzymes, as well as daily variations in the acetylation status of many mitochondrial proteins (Masri et al. 2013). Additionally, in Bmal1 knockout animals, numerous mitochondrial proteins have altered acetylation statuses (Peek et al. 2013; Cela et al. 2016; Mauvoisin et al. 2017). It appears, therefore, that mitochondrial protein acetylation is under circadian clock control, which raises the question of whether additional post-translational modifications, such as phosphorylation, are likewise rhythmic. In the following sections, we will introduce how mitochondrial dynamics and functions, particularly mitochondrial respiration, biogenesis, and morphological dynamics, are regulated by circadian clock. Additionally, we will explore how changes in mitochondrial function impact the molecular clock, elucidating the interconnection between the circadian clock and mitochondria.

### 4.1. Clock-dependent mitochondrial respiration

**Figure 2.**
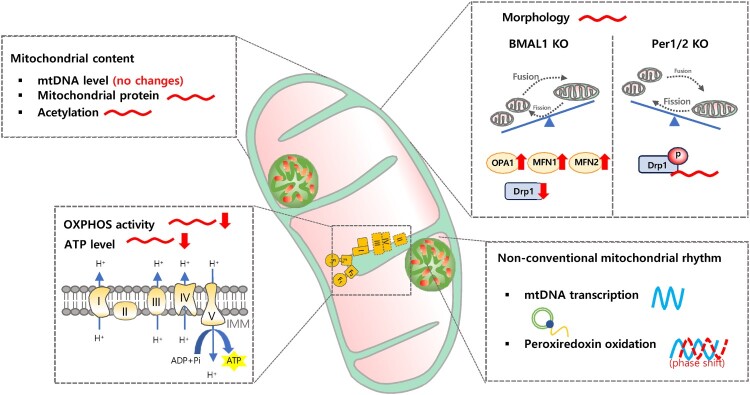
Mitochondrial dysfunction caused by circadian clock malfunction. Circadian clock dysfunction affects various aspects of mitochondrial components, function, and dynamics. In terms of mitochondrial content, circadian clock knockout models showed little impact on mtDNA levels, but they did affect rhythmic mitochondrial protein and rhythmic acetylation profile. Circadian clock dysfunction abolished daily mitochondrial morphological rhythms. In BMAL1 knockout models, mitochondrial fusion-related proteins (OPA1, MFN1, MFN2) increased, while the fission-related protein Drp1 decreased, leading to enlarged swollen mitochondria. However, in PER1/2 knockout models, the rhythm of Drp1 phosphorylation disappeared, resulting in fragmented mitochondria. Despite the differences in morphological changes observed in each circadian clock knockout model, mitochondrial functional aspects such as OXPHOS activity and ATP production levels decreased, with their rhythms also disappearing. Nevertheless, even in the absence of the circadian clock, the 12-hour rhythm of mitochondrial DNA transcription and the rhythmic profile of peroxiredoxin oxidation were maintained, albeit with phase shifts.

Mitochondria utilize nutrients differently throughout the day based on the time of day (Neufeld-Cohen et al. 2016). Carnitine palmitoyltransferase 1 (CPT1), the rate-limiting enzyme in mitochondrial fatty acid uptake, exhibits synchronous rhythmicity in the presence of certain fatty acids, such as palmitoylcarnitine, resulting in rhythmic respiration with peak levels during the transition from the active phase to the rest phase (Neufeld-Cohen et al. 2016). Rhythmic levels and/or phosphorylation of pyruvate dehydrogenase (PDH) likely account for rhythmic pyruvate usage, which similarly peaks later during the rest period (Jacobi et al. 2015). The rhythmic oscillations of these enzymes and respiration rate were abolished in Per1/2 knockout mice, suggesting that the circadian clock protein PERIOD regulates the diurnal utilization of different nutrients to adapt mitochondrial function to energy demand (Neufeld-Cohen et al. 2016). The oxygen consumption rate (OCR) as a readout of mitochondrial respiration, exhibits circadian oscillation (Peek et al. 2013). OCR decreased and diurnal variation was lost in the liver mitochondria of Per1/2 double knockout mice and Bmal1 knockout mice (Peek et al. 2013; Jacobi et al. 2015; Neufeld-Cohen et al. 2016) (Figure 2).

Multiple mechanisms have been shown to control the circadian rhythm of OXPHOS activity. For instance, protein acetylation, which controls mitochondrial function, is associated with the circadian clock-driven NAD+ salvage process by regulating the expression of the NAD+ biosynthesis enzyme NAMPT, particularly during fasting and the cell-autonomous state (Nakahata et al. 2009; Ramsey et al. 2009). Consistently, the NAD+ content, OCR, and fatty acid oxidation are reduced in Bmal1 KO MEF. Furthermore, circadian regulation of mitochondrial OXPHOS is achieved through several pathways: acetylation of mitochondrial proteins by SIRTs (Peek et al. 2013), and via AMPK (Hardie et al. 2012). Circadian clock driven NAD+ dependent SIRT3, a mitochondrial deacetylase, regulates mitochondrial protein acetylation, resulting in controlling the activity of oxidative enzyme and the activity of mitochondrial oxidative enzymes (Peek et al. 2013). AMP-activated protein kinase (AMPK), serine/threonine kinase, is activated and phosphorylated downstream targets upon changes in the ATP-to AMP ratio. AMPK activates PGC1α, which activates mitochondrial biogenesis to produce more ATP (Hardie et al. 2012). Furthermore, AMPK activate mitochondrial fission, and mitophagy by phosphorylation of mitochondrial fission factor (MFF) and ULK1 to maintain mitochondrial quality and sustain cellular ATP-generating capacity (Hardie et al. 2012).

### 4.2. Circadian control of mitochondrial biogenesis

Mitochondrial biogenesis is influenced by various external factors, such as exercise, nutrient supply, and oxidative stress. Mitochondrial contents are commonly assessed through mitochondrial DNA (mtDNA) copy number and mitochondrial protein levels, reflecting the outcomes of mitochondrial biogenesis. Although PGC1α, the master regulator of mitochondrial biogenesis, is rhythmically expressed in mouse liver and skeletal muscle, as well as in synchronous HepG2 cells (Liu et al. 2007; Cela et al. 2016), debates persist regarding whether mitochondrial contents show the circadian rhythm. Jacobi et al. proposed that mtDNA copy number and mitochondrial protein levels measured by MitoTracker exhibit a 24-hour oscillation, which is Bmal1-dependent (Jacobi et al. 2015). However, some studies have suggested the absence of an oscillation in mtDNA copy number and protein levels of respiratory chain complexes and Tomm20, which is mitochondrial outer membrane protein (Peek et al. 2013; van Moorsel et al. 2016). Additionally, mtDNA copy number remains unchanged in circadian clock gene deficient models, including Bmal1 knockout mice in the liver and heart (Peek et al. 2013; Kohsaka et al. 2014), Per2^Brdm1^ mutants, carrying a mutant mPer2 gene with a deletion in the PAS domain, in mouse embryonic fibroblasts (MEF) (Magnone et al. 2015), and Per1/2 knockout mice (Neufeld-Cohen et al. 2016), as well as cardiomyocyte-specific clock mutant mice (Bray et al. 2008).

Although mitochondrial contents have been assessed through mtDNA copy number, discrepancies between mitochondrial contents and mtDNA copy number are observed in circadian clock gene deficient models. Bmal1 deficiency results in the downregulation of PGC1α and decreased mitochondrial protein contents, while mtDNA copy number remains unaffected (Kohsaka et al. 2014). These findings suggest that mtDNA copy number and mitochondrial protein contents may be differentially regulated by circadian clock genes, rendering the measurement of mtDNA copy number as an indicator of mitochondrial contents inaccurate. Furthermore, mitochondrial biogenesis, induced by PGC1α transcriptional activation, is a complex biological process orchestrated by synchronous gene expression of nuclear and mitochondrial genes. Mitochondrial biogenesis involves mtDNA replication, transcription, and the expression of OXPHOS components encoded by nuclear and mitochondrial genomes (Chen et al. 2022). It has been revealed that mitochondrial and cytosolic translation are coordinated by the nuclear genome, rather than at the level of transcription, to facilitate the timely production of OXPHOS complexes during mitochondrial biogenesis (Couvillion et al. 2016). This coordination may also extend to the circadian control of mitochondrial biogenesis. mtDNA copy number results from mtDNA replication and the elimination of damaged mitochondria and mtDNA. Therefore, a more detailed study of the circadian regulation of mitochondrial biogenesis is necessary to understand how the processes involving de novo synthesis of mtDNA and mitochondrial proteins, nuclear-encoded OXPHOS proteins, and the elimination of damaged mtDNA and mitochondria are regulated by the circadian rhythm.

### 4.3. Clock-dependent control of mitochondrial morphological dynamics

Mitochondria are dynamic organelles that constantly undergo fusion and fission, which allows them to adapt to changing energy demands and maintain mitochondrial function. The cycles of mitochondrial fission and fusion regulate mitochondrial morphology and functions, such as mitophagy and energy metabolism. The morphological dynamics of mitochondria coincide with oscillations in ATP content and OXPHOS activity: Mitochondrial fusion induces a tubular network, resulting in an increase in ATP content and high OXPHOS activity, while fragmented forms induced by mitochondrial fission lead to the opposite results (Schmitt et al. 2018). Several reports have shown that mitochondrial morphology displays circadian rhythmicity in serum-shocked human skin fibroblasts and wild-type mice (Jacobi et al. 2015; Schmitt et al. 2018; Xu et al. 2022). However, functional circadian clock deficiency induces abnormal mitochondrial morphological dynamics, resulting in mitochondrial functional impairment (Figure 2). Liver-specific Bmal1 KO mice show an enlarged and swollen mitochondrial phenotype regardless of the time of day, accompanied by decreased expression of mitochondrial fission proteins (Jacobi et al. 2015). These mice also lose diurnal variation in OXPHOS activity, exhibit reduced mitochondrial respiration, and elevated oxidative stress. mPer1/Per2 double-mutant mice exhibit fragmented mitochondrial morphology regardless of the time, accompanied by the disappearance of ATP oscillation and low ATP content (Schmitt et al. 2018). CLOCKΔ19 mice show smaller, more fragmented mitochondrial morphology and lose rhythmic changes in mitochondrial morphology, leading to disordered mitochondrial homeostasis, including diminished mitochondrial respiration rate, ATP level, and elevated ROS levels (Xu et al. 2022).

Mitochondrial fusion and fission are orchestrated by several proteins. Mitochondrial fission is mainly regulated by dynamin-related protein 1 (DRP1), Fis1, and MFF, while mitochondrial fusion is mediated by MFN1, MFN2, and OPA1. ChIP-sequencing of Bmal1, Clock, and Cry (Rey et al. 2011; Koike et al. 2012) has revealed that mitochondria-related genes including Fis1, Bnip3, and Pink1 are bound by Bmal1, Clock, and Cry1 (Jacobi et al. 2015). A recent study has revealed that Drp1 is a key molecule linking the circadian rhythm of mitochondrial morphology to circadian bioenergetics (Schmitt et al. 2018; Xu et al. 2022). The stability of Drp1 mRNA is regulated by CLOCK through its interaction with CLOCK and the RNA-binding protein PUF60 (Xu et al. 2022). Disordered mitochondrial morphological and functional homeostasis observed in CLOCKΔ19 mice is rescued by elevation of Drp1 expression. Furthermore, the post-translational alteration of DRP1 by phosphorylation at Ser637 exhibits a 24-hour rhythm (Schmitt et al. 2018). Suppression of Drp1 abolishes mitochondrial morphological rhythm and ATP oscillation, suggesting that Drp1 plays a central role in the circadian control of mitochondrial architecture and function. The expression of mitochondrial fission proteins, Drp1 and Fis1, and mitophagy proteins, Bnip3 and Pink1, is substantially reduced, and rhythmic expression is abolished in liver-specific Bmal1 knockout mice (Jacobi et al. 2015). Notably, Fis1 overexpression restores mitochondrial morphological and metabolic defects, including reduced ROS levels, normalized uncoupled respiration, and triglyceride contents, as observed in liver-specific Bmal1 knockout mice.

The mRNA expression of mitochondrial fusion proteins, Mfn2 and OPA1, did not change in liver-specific Bmal1 knockout and CLOCKΔ19 mice (Jacobi et al. 2015; Xu et al. 2022). However, the protein level of MFN1, MFN2, and OPA1 is increased in liver-specific Bmal1 knockout mice, suggesting that mitochondrial fusion protein expression is Bmal1-independent and may be post-transcriptionally modulated (Jacobi et al. 2015). It remains an open question how mitochondrial fusion proteins are regulated by the circadian clock and how their circadian modulation impacts mitochondrial homeostasis.

Recent findings have revealed that the mitochondrial fission protein Drp1 plays a crucial role in brain energy homeostasis during sleep (Haynes et al. 2024). The mitochondrial energetic activity of neurons and glial cells regulates the rhythm of lipid accumulation and lipid droplet processing in glial cells, ultimately controlling mitophagy in both neurons and glial cells to maintain healthy mitochondria through the sleep-wake cycle.

### 4.4. Feedback from mitochondria to circadian clock

Changes in mitochondrial metabolism can serve as retrograde signals to the molecular clock. Pharmacological inhibition of OXPHOS and mtDNA depletion induce dramatic disruptions of circadian clock gene expression (Scrima et al. 2016). The mitochondrial NAD+ pool is maintained separately from the nuclear/cytoplasmic NAD+ pool, and their pools are intricately connected by NAD+/NADH redox shuttles and NAD+ biosynthetic pathways in each subcellular compartment (Stein and Imai 2012). Cellular NAD+ levels oscillate in a diurnal manner and couple cellular metabolism between mitochondria and the molecular clock. Circadian control of NAD+ levels modulates mitochondrial oxidative function through the control of SIRT3 activity and mitochondrial protein acetylation (Peek et al. 2013). NAD+ also directly influences the molecular clock system by regulating SIRT1 activity. NAD+-dependent SIRT1 binds CLOCK-BMAL1 in a diurnal manner and induces the deacetylation and degradation of Per2, leading to high-amplitude circadian clock gene transcription (Asher et al. 2008). Furthermore, SIRT1 activity regulates the circadian cycle by functioning as a histone deacetylase. SIRT1 directly interacts with CLOCK and induces chromatin remodeling (Nakahata et al. 2008). High AMP/ATP levels activate AMPK (Mihaylova and Shaw 2011). AMPK acts as an energy sensor linking nutrient signals to the circadian clock. AMPK activation regulates the circadian rhythm through the phosphorylation of Cry1 and destabilization of Cry1 by reducing its interaction with Per2 and increasing its binding to FBXL3, a ubiquitin ligase (Lamia et al. 2009). Notably, suppression of Drp1-mediated mitochondrial fission eliminates circadian ATP production and increases the circadian period length of Bmal1 expression, suggesting that Drp1-mediated mitochondrial ATP rhythm feeds back to the core circadian oscillator (Schmitt et al. 2018).

Mitochondrial-driven ion homeostasis may be important for molecular clock gene expression. Leucine zipper-EF-hand-containing transmembrane protein 1 (Letm1) is located in the mitochondrial inner membrane and functions as a mitochondrial Ca2+/H+ antiporter. Letm1 knockdown lengthens the free-running period of the circadian locomotor rhythm and suppresses the circadian rhythm of cytosolic H+ levels in lateral neurons, the circadian pacemaker neurons in Drosophila, and dampens circadian cytosolic Ca2+ and the rhythm of Bmal1 transcription specifically in the SCN, suggesting that mitochondrial LETM1-driven robust ionic rhythms feedback to TTFLs (Morioka et al. 2022).

## 5. Non-conventional mitochondrial rhythm

There is evidence suggesting that mitochondrial oscillation operates independently of the conventional nuclear-derived circadian clock. In a study by Ray et al., Bmal1 knockout mice displayed a 24-hour oscillation of the transcriptome, proteome, and phosphoproteome synchronized with a pulse of the glucocorticoid hormone dexamethasone in liver and fibroblast cells (Ray et al. 2020). The non-canonical oscillations identified in Bmal1 knockout persist in the absence of exogenous cues such as light or temperature. The molecular mechanism driving rhythmicity in Bmal1 knockout tissues suggests that transcriptional regulation of multiple E26 transformation-specific (ETS) factors and non-transcriptionally regulated peroxiredoxin (RPDX) oxidation could drive these oscillations in Bmal1 knockout systems. Interestingly, in the gene ontology enrichment analysis of rhythmic proteome and phosphoproteome in the absence of BMAL1, cellular metabolism and oxidation-reduction were highlighted as significant biological processes, with mitochondria emerging as prominent cellular components. Given that the ETS transcription factor exhibits a potent inhibitory effect when co-expressed with the CLOCK-BMAL1-CRY complex (Kondratov et al. 2006), and the PRDX oxidation rhythm persists in its rhythmic profile, albeit with a phase shift, in circadian clock mutants (Edgar et al. 2012) (Figure 2), there is a possibility that mitochondrial intrinsic oscillations are suppressed or operate independently of the circadian clock. However, it remains to be elucidated whether mitochondrial intrinsic rhythm exists or not.

Interestingly, the transcription and translation of mtDNA-encoded genes from both the heavy and light strands of mtDNA exhibit a 12-hour rhythm, and nuclear-encoded genes comprising the electron transport chain also display a 12-hour rhythm. The 12-hour rhythm of mtDNA transcription is evolutionarily conserved (Zhu et al. 2017). Mitochondrial transcription factors B1 and B2 (Tfb1m and Tfab2m) also exhibit a 12-hour rhythm (Zhu et al. 2017). The 12-hour pacemaker is thought to be independent of the circadian clock because cell-autonomous 12-hour rhythms are synchronized by metabolic and ER stress cues, not by circadian clock-synchronizing cues (Zhu et al. 2017) (Figure 2). However, the experimental evidence regarding whether this rhythmic mitochondrial transcription and translation are maintained in Bmal1 knockout or other circadian rhythm-controlling gene knockouts is missing. It is still necessary to clarify the physiological significance of 12-hour rhythmicity and the molecular mechanism of mitochondrial-related 12-hour rhythm. The OXPHOS complex is comprised of both mtDNA-encoded proteins and nuclear-encoded proteins. Remaining questions include whether nuclear-encoded proteins exhibit rhythmicity and, if they have a 24-hour rhythmicity, how they coordinate their protein abundance with mtDNA-encoded proteins to compromise the OXPHOS complex.

It is well established that there is coupling between the circadian oscillator and redox systems, where the molecular clock directly regulates the levels of redox metabolites and antioxidant enzymes (Milev and Reddy 2015). One of these antioxidant enzymes, Peroxiredoxin, is an evolutionarily highly conserved protein and exhibits circadian oscillation. Interestingly, it has been observed that peroxiredoxin exhibits a circadian rhythm in human red blood cells lacking nuclei (O’Neill and Reddy 2011). This demonstrates the existence of non-transcriptional circadian oscillation independent of transcription. Considering that the circadian rhythm in prokaryotes is regulated by post-translational mechanisms and that the function of peroxiredoxin is closely linked to mitochondrial oxidative function, it is possible that it is linked to the intrinsic rhythm of mitochondria.

### 5.1. Perspectives

The circadian clock is a phenomenon repeatedly found across domains of life, from bacteria to mammals, although its driving molecular mechanism is independently evolved. Considering that mitochondria evolved from alpha prokaryotes, it is not surprising that mitochondria have an independent circadian rhythm. Given that the circadian system of bacteria responds to metabolic changes rather than the light-dark cycle per se, and metabolic activity serves as the main synchronizer of their clocks (Pattanayak et al. 2015), it is highly probable that the intrinsic rhythm of mitochondria is closely associated with metabolic cues. Therefore, it can be considered that the circadian clock in the nucleus and the metabolic clock in mitochondria have evolved through mutual interaction. Further studies are required to examine the existence of mitochondrial intrinsic rhythm in various mitochondrial biological processes. Additionally, more research is needed to investigate how this intrinsic rhythm is tuned by the circadian clock in response to changes in metabolic activity.

Mitochondrial function regulated by the circadian clock influences various behaviors and physiological processes within our bodies. The circadian clock is associated with various pathological conditions, such as aging, neuropsychiatric diseases, neurodegeneration, and metabolic disorders. Mitochondrial dysfunction also contributes to a variety of diseases. Accordingly, several reports suggest that dysregulation of mitochondrial function is found in circadian rhythm-related diseases (Ulgherait et al. 2020; Gabriel et al. 2021). For instance, circadian clock dysregulation in primary myocytes from individuals and skeletal muscle biopsies with type 2 diabetes is linked to rhythmic mitochondrial respiration and rhythmic transcripts of the mitochondrial inner membrane (Gabriel et al. 2021). Circadian clock components, Period and Timeless in Drosophila, affect lifespan determination by altering cellular respiration via mitochondrial uncoupling (Ulgherait et al. 2020), suggesting that the mitochondiral metabolism and circadian rythms are coupled at whole systemic level. From this perspective, it is conceivable that pathological conditions may disrupt the coordination of these two rhythms, causing them to go off-beat, potentially leading to adverse effects. Therefore, understanding the on- and off-beat of mitochondria and circadian rhythms from a chronobiological perspective can offer deeper insights into various physiological and pathological states.
